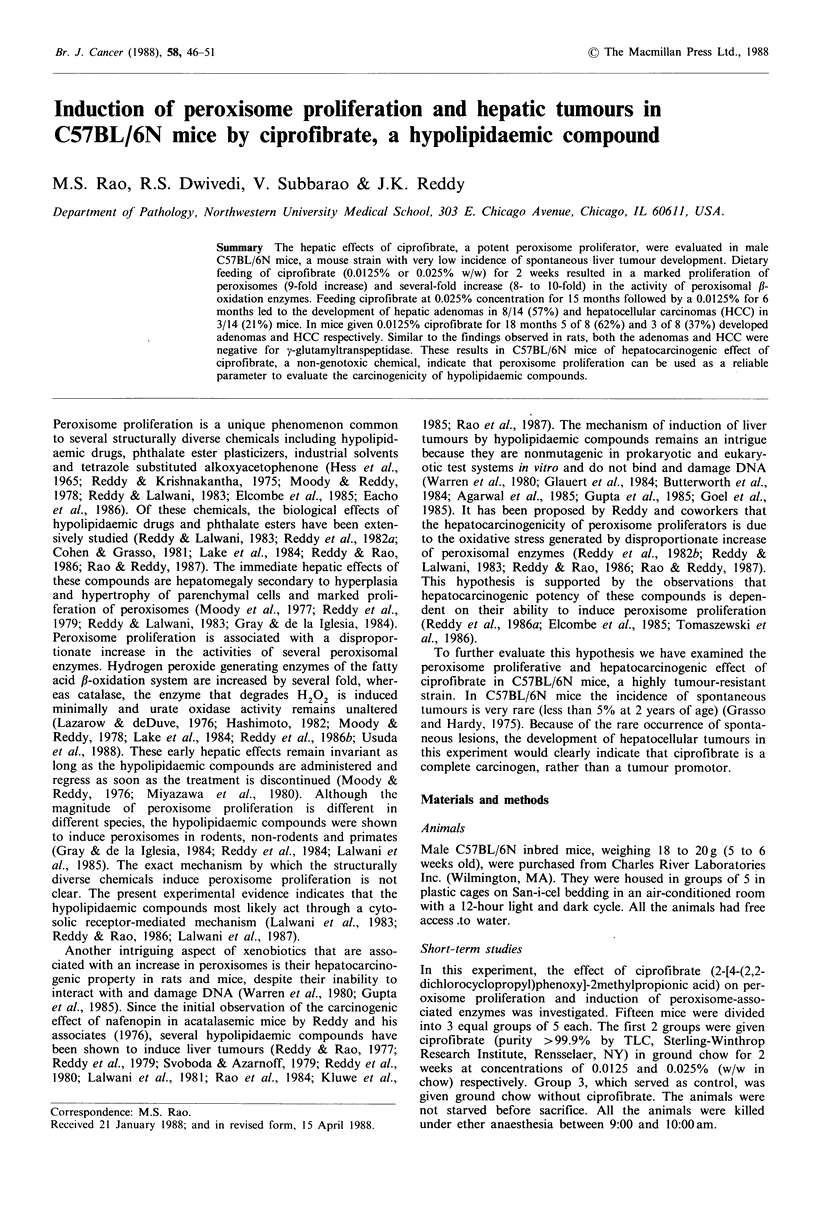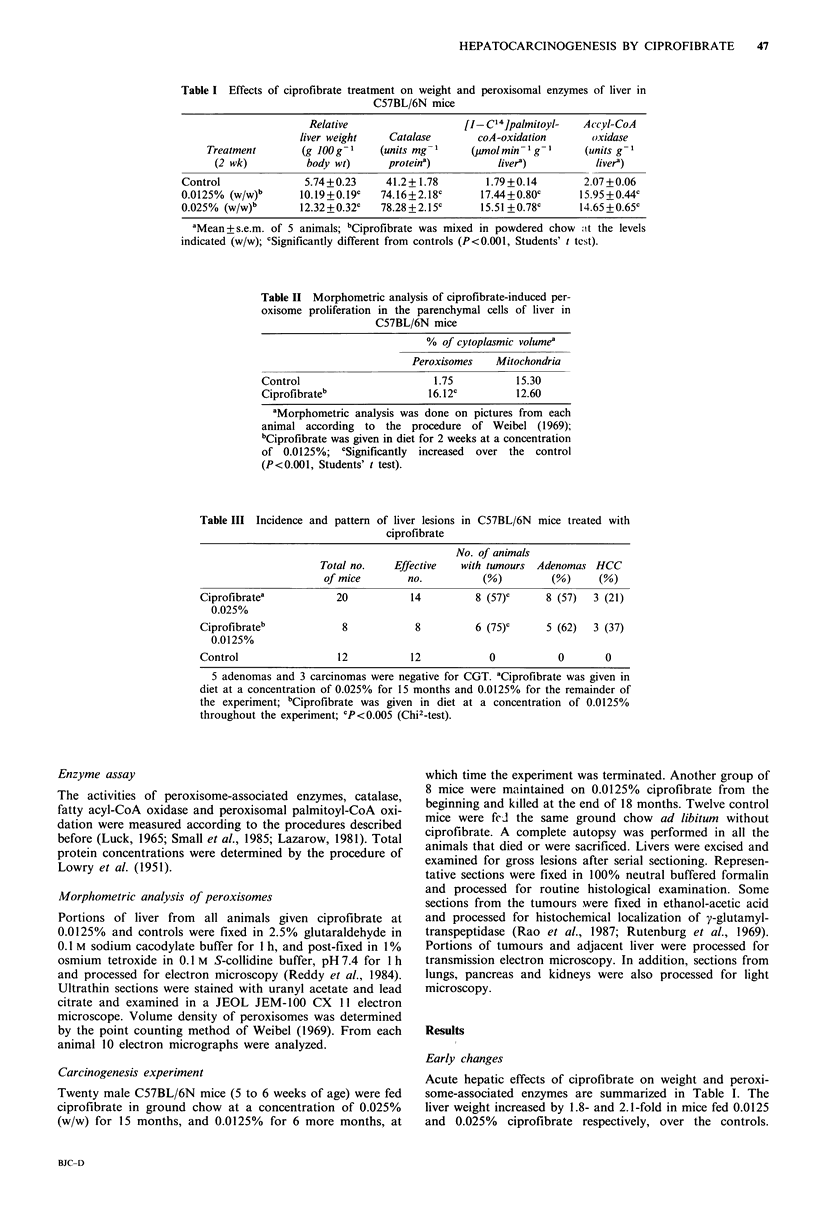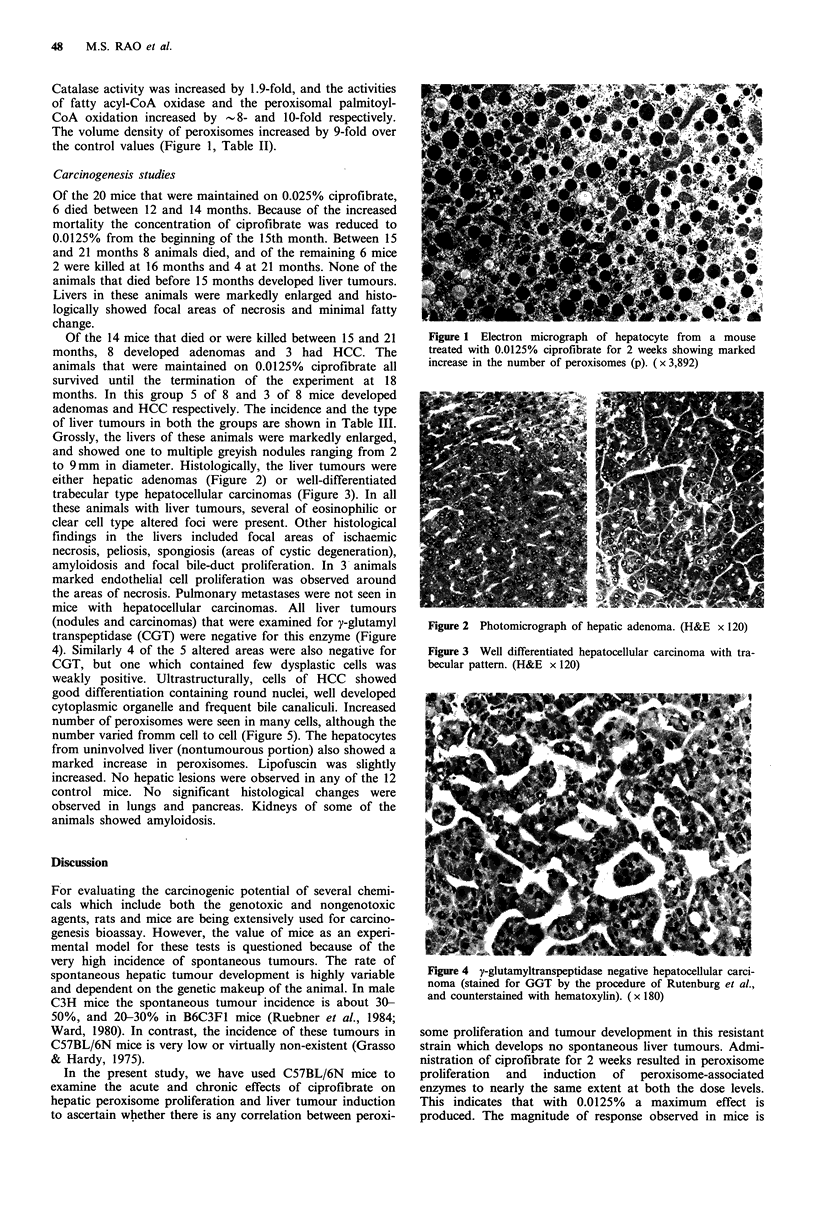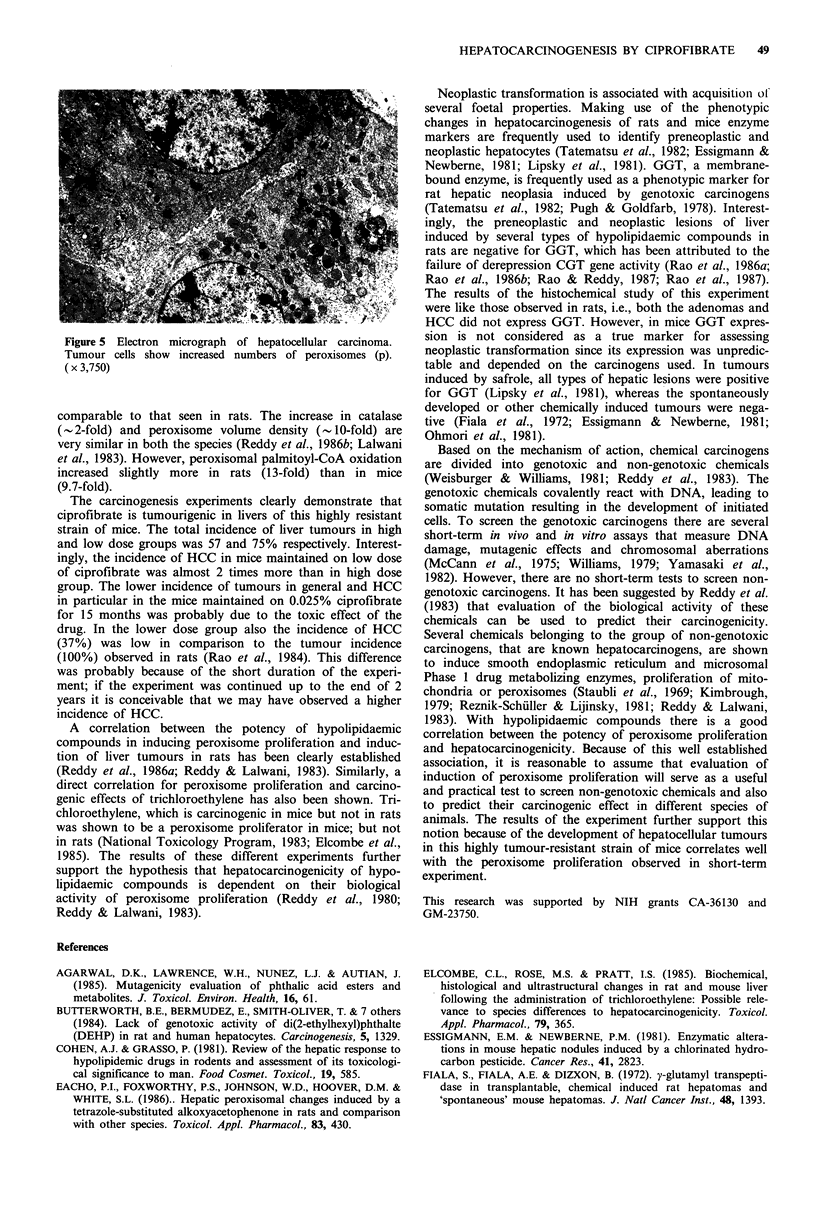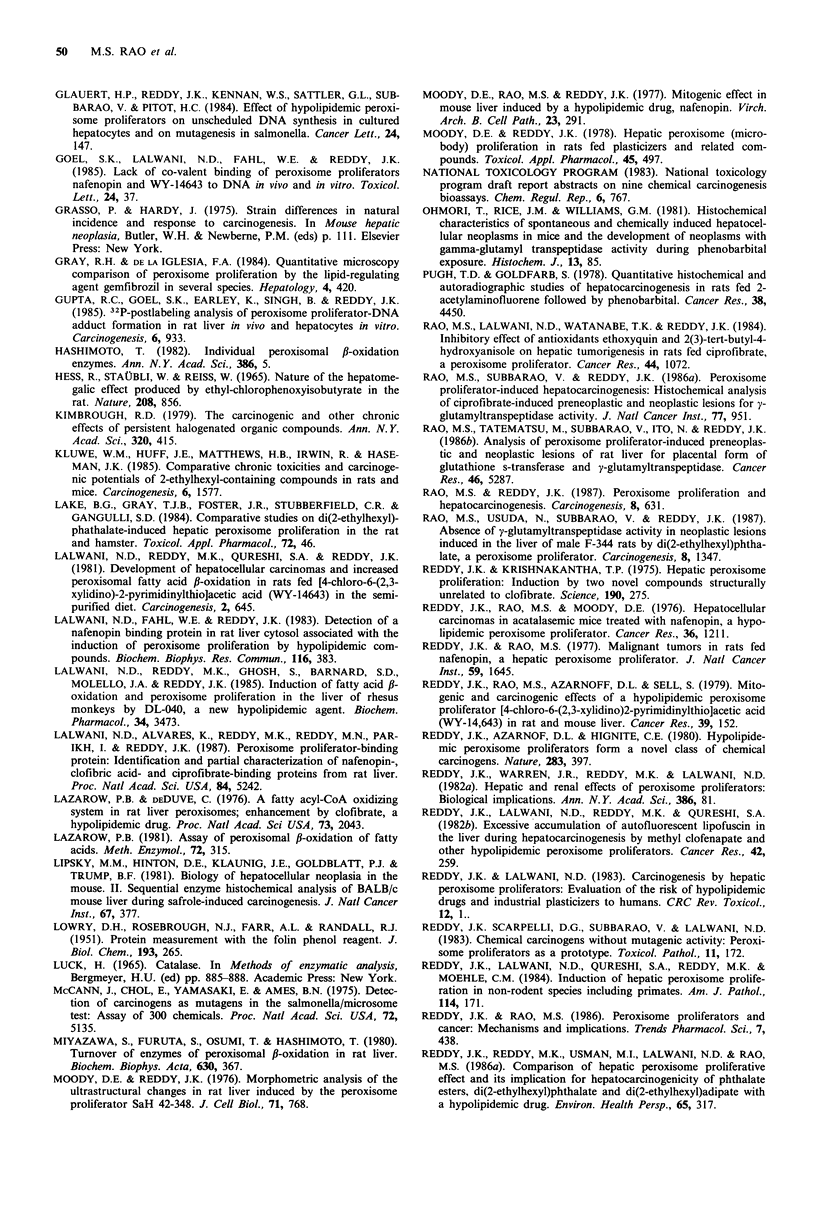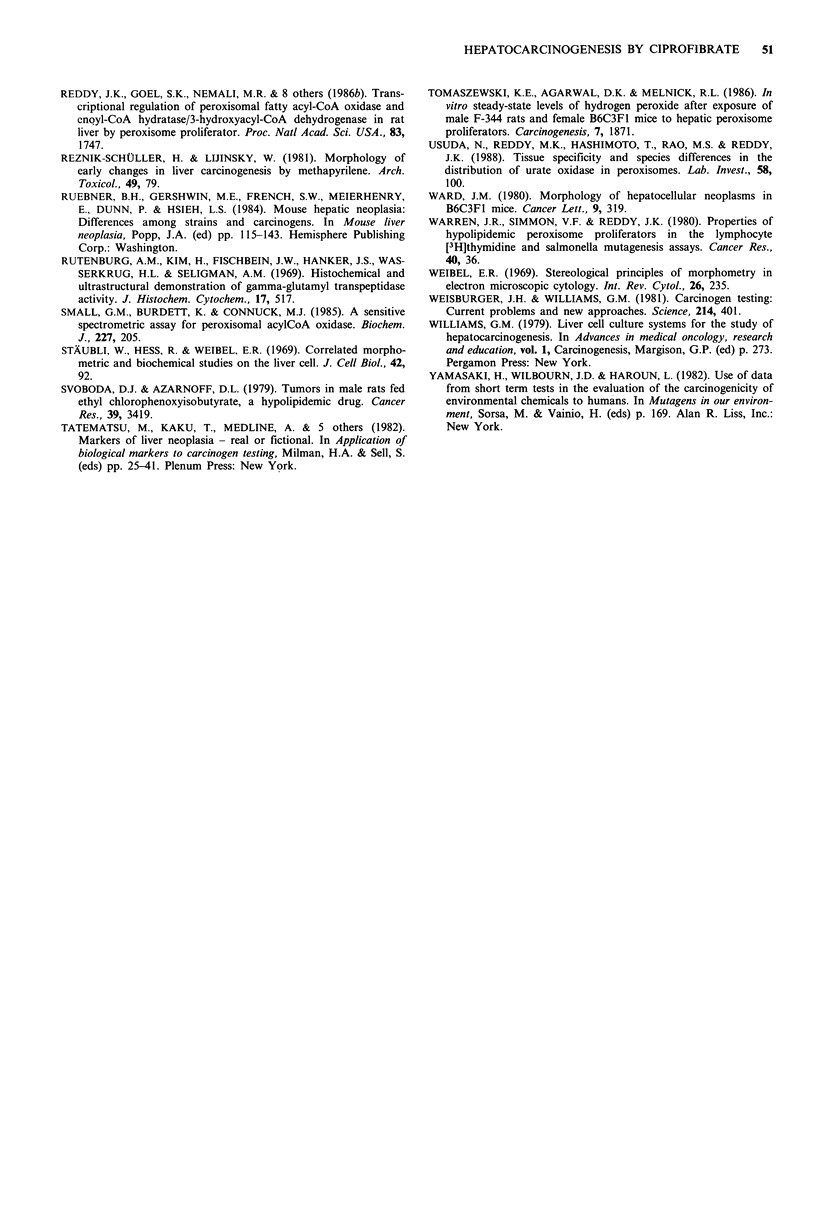# Induction of peroxisome proliferation and hepatic tumours in C57BL/6N mice by ciprofibrate, a hypolipidaemic compound.

**DOI:** 10.1038/bjc.1988.159

**Published:** 1988-07

**Authors:** M. S. Rao, R. S. Dwivedi, V. Subbarao, J. K. Reddy

**Affiliations:** Department of Pathology, Northwestern University Medical School, Chicago, IL 60611.

## Abstract

**Images:**


*Br. J. Cancer* (1988), **58**, 46–51 © The Macmillan Press Ltd., 1988

### Induction of peroxisome proliferation and hepatic tumours in C57BL/6N mice by ciprofibrate, a hypolipidaemic compound

M.S. Rao, R.S. Dwivedi, V. Subbarao & J.K. Reddy

*Department of Pathology, Northwestern University Medical School, 303 E. Chicago Avenue, Chicago, IL 60611, USA.*

**Summary** The hepatic effects of ciprofibrate, a potent peroxisome proliferator, were evaluated in male C57BL/6N mice, a mouse strain with very low incidence of spontaneous liver tumour development. Dietary feeding of ciprofibrate (0.0125% or 0.025% w/w) for 2 weeks resulted in a marked proliferation of peroxisomes (9-fold increase) and several-fold increase (8- to 10-fold) in the activity of peroxisomal $\beta$-oxidation enzymes. Feeding ciprofibrate at 0.025% concentration for 15 months followed by a 0.0125% for 6 months led to the development of hepatic adenomas in 8/14 (57%) and hepatocellular carcinomas (HCC) in 3/14 (21%) mice. In mice given 0.0125% ciprofibrate for 18 months 5 of 8 (62%) and 3 of 8 (37%) developed adenomas and HCC respectively. Similar to the findings observed in rats, both the adenomas and HCC were negative for $\gamma$-glutamyltranspeptidase. These results in C57BL/6N mice of hepatocarcinogenic effect of ciprofibrate, a non-genotoxic chemical, indicate that peroxisome proliferation can be used as a reliable parameter to evaluate the carcinogenicity of hypolipidaemic compounds.

Peroxisome proliferation is a unique phenomenon common to several structurally diverse chemicals including hypolipidaemic drugs, phthalate ester plasticizers, industrial solvents and tetrazole substituted alkoxyacetophenone (Hess *et al.*, 1965; Reddy & Krishnakantha, 1975; Moody & Reddy, 1978; Reddy & Lalwani, 1983; Elcombe *et al.*, 1985; Eacho *et al.*, 1986). Of these chemicals, the biological effects of hypolipidaemic drugs and phthalate esters have been extensively studied (Reddy & Lalwani, 1983; Reddy *et al.*, 1982*a*; Cohen & Grasso, 1981; Lake *et al.*, 1984; Reddy & Rao, 1986; Rao & Reddy, 1987). The immediate hepatic effects of these compounds are hepatomegaly secondary to hyperplasia and hypertrophy of parenchymal cells and marked proliferation of peroxisomes (Moody *et al.*, 1977; Reddy *et al.*, 1979; Reddy & Lalwani, 1983; Gray & de la Iglesia, 1984). Peroxisome proliferation is associated with a disproportionate increase in the activities of several peroxisomal enzymes. Hydrogen peroxide generating enzymes of the fatty acid $\beta$-oxidation system are increased by several fold, whereas catalase, the enzyme that degrades $H_2O_2$ is induced minimally and urate oxidase activity remains unaltered (Lazarow & deDuve, 1976; Hashimoto, 1982; Moody & Reddy, 1978; Lake *et al.*, 1984; Reddy *et al.*, 1986*b*; Usuda *et al.*, 1988). These early hepatic effects remain invariant as long as the hypolipidaemic compounds are administered and regress as soon as the treatment is discontinued (Moody & Reddy, 1976; Miyazawa *et al.*, 1980). Although the magnitude of peroxisome proliferation is different in different species, the hypolipidaemic compounds were shown to induce peroxisomes in rodents, non-rodents and primates (Gray & de la Iglesia, 1984; Reddy *et al.*, 1984; Lalwani *et al.*, 1985). The exact mechanism by which the structurally diverse chemicals induce peroxisome proliferation is not clear. The present experimental evidence indicates that the hypolipidaemic compounds most likely act through a cytosolic receptor-mediated mechanism (Lalwani *et al.*, 1983; Reddy & Rao, 1986; Lalwani *et al.*, 1987).

Another intriguing aspect of xenobiotics that are associated with an increase in peroxisomes is their hepatocarcinogenic property in rats and mice, despite their inability to interact with and damage DNA (Warren *et al.*, 1980; Gupta *et al.*, 1985). Since the initial observation of the carcinogenic effect of nafenopin in acatalasemic mice by Reddy and his associates (1976), several hypolipidaemic compounds have been shown to induce liver tumours (Reddy & Rao, 1977; Reddy *et al.*, 1979; Svoboda & Azarnoff, 1979; Reddy *et al.*, 1980; Lalwani *et al.*, 1981; Rao *et al.*, 1984; Kluwe *et al.*, 1985; Rao *et al.*, 1987). The mechanism of induction of liver tumours by hypolipidaemic compounds remains an intrigue because they are nonmutagenic in prokaryotic and eukaryotic test systems *in vitro* and do not bind and damage DNA (Warren *et al.*, 1980; Glauert *et al.*, 1984; Butterworth *et al.*, 1984; Agarwal *et al.*, 1985; Gupta *et al.*, 1985; Goel *et al.*, 1985). It has been proposed by Reddy and coworkers that the hepatocarcinogenicity of peroxisome proliferators is due to the oxidative stress generated by disproportionate increase of peroxisomal enzymes (Reddy *et al.*, 1982*b*; Reddy & Lalwani, 1983; Reddy & Rao, 1986; Rao & Reddy, 1987). This hypothesis is supported by the observations that hepatocarcinogenic potency of these compounds is dependent on their ability to induce peroxisome proliferation (Reddy *et al.*, 1986*a*; Elcombe *et al.*, 1985; Tomaszewski *et al.*, 1986).

To further evaluate this hypothesis we have examined the peroxisome proliferative and hepatocarcinogenic effect of ciprofibrate in C57BL/6N mice, a highly tumour-resistant strain. In C57BL/6N mice the incidence of spontaneous tumours is very rare (less than 5% at 2 years of age) (Grasso and Hardy, 1975). Because of the rare occurrence of spontaneous lesions, the development of hepatocellular tumours in this experiment would clearly indicate that ciprofibrate is a complete carcinogen, rather than a tumour promotor.

### Materials and methods

### *Animals*

Male C57BL/6N inbred mice, weighing 18 to 20 g (5 to 6 weeks old), were purchased from Charles River Laboratories Inc. (Wilmington, MA). They were housed in groups of 5 in plastic cages on San-i-cel bedding in an air-conditioned room with a 12-hour light and dark cycle. All the animals had free access .to water.

### *Short-term studies*

In this experiment, the effect of ciprofibrate (2-[4-(2,2-dichlorocyclopropyl)phenoxy]-2methylpropionic acid) on peroxisome proliferation and induction of peroxisome-associated enzymes was investigated. Fifteen mice were divided into 3 equal groups of 5 each. The first 2 groups were given ciprofibrate (purity >99.9% by TLC, Sterling-Winthrop Research Institute, Rensselaer, NY) in ground chow for 2 weeks at concentrations of 0.0125 and 0.025% (w/w in chow) respectively. Group 3, which served as control, was given ground chow without ciprofibrate. The animals were not starved before sacrifice. All the animals were killed under ether anaesthesia between 9:00 and 10:00 am.

Correspondence: M.S. Rao.
Received 21 January 1988; and in revised form, 15 April 1988.

**Table I** Effects of ciprofibrate treatment on weight and peroxisomal enzymes of liver in C57BL/6N mice

| *Treatment (2 wk)* | *Relative liver weight (g 100 g$^{-1}$ body wt)* | *Catalase (units mg$^{-1}$ protein*[a]*)* | *[1–C$^{14}$]palmitoyl-coA-oxidation (μmol min$^{-1}$ g$^{-1}$ liver*[a]*)* | *Accyl-CoA oxidase (units g$^{-1}$ liver*[a]*)* |
|---|---|---|---|---|
| Control | 5.74±0.23 | 41.2±1.78 | 1.79±0.14 | 2.07±0.06 |
| 0.0125% (w/w)[b] | 10.19±0.19[c] | 74.16±2.18[c] | 17.44±0.80[c] | 15.95±0.44[c] |
| 0.025% (w/w)[b] | 12.32±0.32[c] | 78.28±2.15[c] | 15.51±0.78[c] | 14.65±0.65[c] |

[a]Mean±s.e.m. of 5 animals; [b]Ciprofibrate was mixed in powdered chow at the levels indicated (w/w); [c]Significantly different from controls ($P<0.001$, Students' $t$ test).

**Table II** Morphometric analysis of ciprofibrate-induced peroxisome proliferation in the parenchymal cells of liver in C57BL/6N mice

| | *% of cytoplasmic volume*[a] | |
|---|---|---|
| | *Peroxisomes* | *Mitochondria* |
| Control | 1.75 | 15.30 |
| Ciprofibrate[b] | 16.12[c] | 12.60 |

[a]Morphometric analysis was done on pictures from each animal according to the procedure of Weibel (1969); [b]Ciprofibrate was given in diet for 2 weeks at a concentration of 0.0125%; [c]Significantly increased over the control ($P<0.001$, Students' $t$ test).

**Table III** Incidence and pattern of liver lesions in C57BL/6N mice treated with ciprofibrate

| | *Total no. of mice* | *Effective no.* | *No. of animals with tumours (%)* | *Adenomas (%)* | *HCC (%)* |
|---|---|---|---|---|---|
| Ciprofibrate[a] 0.025% | 20 | 14 | 8 (57)[c] | 8 (57) | 3 (21) |
| Ciprofibrate[b] 0.0125% | 8 | 8 | 6 (75)[c] | 5 (62) | 3 (37) |
| Control | 12 | 12 | 0 | 0 | 0 |

5 adenomas and 3 carcinomas were negative for CGT. [a]Ciprofibrate was given in diet at a concentration of 0.025% for 15 months and 0.0125% for the remainder of the experiment; [b]Ciprofibrate was given in diet at a concentration of 0.0125% throughout the experiment; [c]$P<0.005$ (Chi$^2$-test).

### *Enzyme assay*

The activities of peroxisome-associated enzymes, catalase, fatty acyl-CoA oxidase and peroxisomal palmitoyl-CoA oxidation were measured according to the procedures described before (Luck, 1965; Small *et al.*, 1985; Lazarow, 1981). Total protein concentrations were determined by the procedure of Lowry *et al.* (1951).

### *Morphometric analysis of peroxisomes*

Portions of liver from all animals given ciprofibrate at 0.0125% and controls were fixed in 2.5% glutaraldehyde in 0.1 M sodium cacodylate buffer for 1 h, and post-fixed in 1% osmium tetroxide in 0.1 M *S*-collidine buffer, pH 7.4 for 1 h and processed for electron microscopy (Reddy *et al.*, 1984). Ultrathin sections were stained with uranyl acetate and lead citrate and examined in a JEOL JEM-100 CX 11 electron microscope. Volume density of peroxisomes was determined by the point counting method of Weibel (1969). From each animal 10 electron micrographs were analyzed.

### *Carcinogenesis experiment*

Twenty male C57BL/6N mice (5 to 6 weeks of age) were fed ciprofibrate in ground chow at a concentration of 0.025% (w/w) for 15 months, and 0.0125% for 6 more months, at which time the experiment was terminated. Another group of 8 mice were maintained on 0.0125% ciprofibrate from the beginning and killed at the end of 18 months. Twelve control mice were fed the same ground chow *ad libitum* without ciprofibrate. A complete autopsy was performed in all the animals that died or were sacrificed. Livers were excised and examined for gross lesions after serial sectioning. Representative sections were fixed in 100% neutral buffered formalin and processed for routine histological examination. Some sections from the tumours were fixed in ethanol-acetic acid and processed for histochemical localization of γ-glutamyltranspeptidase (Rao *et al.*, 1987; Rutenburg *et al.*, 1969). Portions of tumours and adjacent liver were processed for transmission electron microscopy. In addition, sections from lungs, pancreas and kidneys were also processed for light microscopy.

### Results

### *Early changes*

Acute hepatic effects of ciprofibrate on weight and peroxisome-associated enzymes are summarized in Table I. The liver weight increased by 1.8- and 2.1-fold in mice fed 0.0125 and 0.025% ciprofibrate respectively, over the controls.

Catalase activity was increased by 1.9-fold, and the activities of fatty acyl-CoA oxidase and the peroxisomal palmitoyl-CoA oxidation increased by ~8- and 10-fold respectively. The volume density of peroxisomes increased by 9-fold over the control values (Figure 1, Table II).

*Carcinogenesis studies*

Of the 20 mice that were maintained on 0.025% ciprofibrate, 6 died between 12 and 14 months. Because of the increased mortality the concentration of ciprofibrate was reduced to 0.0125% from the beginning of the 15th month. Between 15 and 21 months 8 animals died, and of the remaining 6 mice 2 were killed at 16 months and 4 at 21 months. None of the animals that died before 15 months developed liver tumours. Livers in these animals were markedly enlarged and histologically showed focal areas of necrosis and minimal fatty change.

Of the 14 mice that died or were killed between 15 and 21 months, 8 developed adenomas and 3 had HCC. The animals that were maintained on 0.0125% ciprofibrate all survived until the termination of the experiment at 18 months. In this group 5 of 8 and 3 of 8 mice developed adenomas and HCC respectively. The incidence and the type of liver tumours in both the groups are shown in Table III. Grossly, the livers of these animals were markedly enlarged, and showed one to multiple greyish nodules ranging from 2 to 9 mm in diameter. Histologically, the liver tumours were either hepatic adenomas (Figure 2) or well-differentiated trabecular type hepatocellular carcinomas (Figure 3). In all these animals with liver tumours, several of eosinophilic or clear cell type altered foci were present. Other histological findings in the livers included focal areas of ischaemic necrosis, peliosis, spongiosis (areas of cystic degeneration), amyloidosis and focal bile-duct proliferation. In 3 animals marked endothelial cell proliferation was observed around the areas of necrosis. Pulmonary metastases were not seen in mice with hepatocellular carcinomas. All liver tumours (nodules and carcinomas) that were examined for γ-glutamyl transpeptidase (CGT) were negative for this enzyme (Figure 4). Similarly 4 of the 5 altered areas were also negative for CGT, but one which contained few dysplastic cells was weakly positive. Ultrastructurally, cells of HCC showed good differentiation containing round nuclei, well developed cytoplasmic organelle and frequent bile canaliculi. Increased number of peroxisomes were seen in many cells, although the number varied fromm cell to cell (Figure 5). The hepatocytes from uninvolved liver (nontumourous portion) also showed a marked increase in peroxisomes. Lipofuscin was slightly increased. No hepatic lesions were observed in any of the 12 control mice. No significant histological changes were observed in lungs and pancreas. Kidneys of some of the animals showed amyloidosis.

**Figure 1** Electron micrograph of hepatocyte from a mouse treated with 0.0125% ciprofibrate for 2 weeks showing marked increase in the number of peroxisomes (p). ( × 3,892)

**Figure 2** Photomicrograph of hepatic adenoma. (H&E × 120)

**Figure 3** Well differentiated hepatocellular carcinoma with trabecular pattern. (H&E × 120)

**Figure 4** γ-glutamyltranspeptidase negative hepatocellular carcinoma (stained for GGT by the procedure of Rutenburg *et al.*, and counterstained with hematoxylin). ( × 180)

### Discussion

For evaluating the carcinogenic potential of several chemicals which include both the genotoxic and nongenotoxic agents, rats and mice are being extensively used for carcinogenesis bioassay. However, the value of mice as an experimental model for these tests is questioned because of the very high incidence of spontaneous tumours. The rate of spontaneous hepatic tumour development is highly variable and dependent on the genetic makeup of the animal. In male C3H mice the spontaneous tumour incidence is about 30–50%, and 20–30% in B6C3F1 mice (Ruebner *et al.*, 1984; Ward, 1980). In contrast, the incidence of these tumours in C57BL/6N mice is very low or virtually non-existent (Grasso & Hardy, 1975).

In the present study, we have used C57BL/6N mice to examine the acute and chronic effects of ciprofibrate on hepatic peroxisome proliferation and liver tumour induction to ascertain whether there is any correlation between peroxisome proliferation and tumour development in this resistant strain which develops no spontaneous liver tumours. Administration of ciprofibrate for 2 weeks resulted in peroxisome proliferation and induction of peroxisome-associated enzymes to nearly the same extent at both the dose levels. This indicates that with 0.0125% a maximum effect is produced. The magnitude of response observed in mice is

**Figure 5** Electron micrograph of hepatocellular carcinoma. Tumour cells show increased numbers of peroxisomes (p). (×3,750)

comparable to that seen in rats. The increase in catalase (~2-fold) and peroxisome volume density (~10-fold) are very similar in both the species (Reddy *et al.*, 1986*b*; Lalwani *et al.*, 1983). However, peroxisomal palmitoyl-CoA oxidation increased slightly more in rats (13-fold) than in mice (9.7-fold).

The carcinogenesis experiments clearly demonstrate that ciprofibrate is tumourigenic in livers of this highly resistant strain of mice. The total incidence of liver tumours in high and low dose groups was 57 and 75% respectively. Interestingly, the incidence of HCC in mice maintained on low dose of ciprofibrate was almost 2 times more than in high dose group. The lower incidence of tumours in general and HCC in particular in the mice maintained on 0.025% ciprofibrate for 15 months was probably due to the toxic effect of the drug. In the lower dose group also the incidence of HCC (37%) was low in comparison to the tumour incidence (100%) observed in rats (Rao *et al.*, 1984). This difference was probably because of the short duration of the experiment; if the experiment was continued up to the end of 2 years it is conceivable that we may have observed a higher incidence of HCC.

A correlation between the potency of hypolipidaemic compounds in inducing peroxisome proliferation and induction of liver tumours in rats has been clearly established (Reddy *et al.*, 1986*a*; Reddy & Lalwani, 1983). Similarly, a direct correlation for peroxisome proliferation and carcinogenic effects of trichloroethylene has also been shown. Trichloroethylene, which is carcinogenic in mice but not in rats was shown to be a peroxisome proliferator in mice; but not in rats (National Toxicology Program, 1983; Elcombe *et al.*, 1985). The results of these different experiments further support the hypothesis that hepatocarcinogenicity of hypolipidaemic compounds is dependent on their biological activity of peroxisome proliferation (Reddy *et al.*, 1980; Reddy & Lalwani, 1983).

Neoplastic transformation is associated with acquisition of several foetal properties. Making use of the phenotypic changes in hepatocarcinogenesis of rats and mice enzyme markers are frequently used to identify preneoplastic and neoplastic hepatocytes (Tatematsu *et al.*, 1982; Essigmann & Newberne, 1981; Lipsky *et al.*, 1981). GGT, a membrane-bound enzyme, is frequently used as a phenotypic marker for rat hepatic neoplasia induced by genotoxic carcinogens (Tatematsu *et al.*, 1982; Pugh & Goldfarb, 1978). Interestingly, the preneoplastic and neoplastic lesions of liver induced by several types of hypolipidaemic compounds in rats are negative for GGT, which has been attributed to the failure of derepression CGT gene activity (Rao *et al.*, 1986*a*; Rao *et al.*, 1986*b*; Rao & Reddy, 1987; Rao *et al.*, 1987). The results of the histochemical study of this experiment were like those observed in rats, i.e., both the adenomas and HCC did not express GGT. However, in mice GGT expression is not considered as a true marker for assessing neoplastic transformation since its expression was unpredictable and depended on the carcinogens used. In tumours induced by safrole, all types of hepatic lesions were positive for GGT (Lipsky *et al.*, 1981), whereas the spontaneously developed or other chemically induced tumours were negative (Fiala *et al.*, 1972; Essigmann & Newberne, 1981; Ohmori *et al.*, 1981).

Based on the mechanism of action, chemical carcinogens are divided into genotoxic and non-genotoxic chemicals (Weisburger & Williams, 1981; Reddy *et al.*, 1983). The genotoxic chemicals covalently react with DNA, leading to somatic mutation resulting in the development of initiated cells. To screen the genotoxic carcinogens there are several short-term *in vivo* and *in vitro* assays that measure DNA damage, mutagenic effects and chromosomal aberrations (McCann *et al.*, 1975; Williams, 1979; Yamasaki *et al.*, 1982). However, there are no short-term tests to screen non-genotoxic carcinogens. It has been suggested by Reddy *et al.* (1983) that evaluation of the biological activity of these chemicals can be used to predict their carcinogenicity. Several chemicals belonging to the group of non-genotoxic carcinogens, that are known hepatocarcinogens, are shown to induce smooth endoplasmic reticulum and microsomal Phase 1 drug metabolizing enzymes, proliferation of mitochondria or peroxisomes (Staubli *et al.*, 1969; Kimbrough, 1979; Reznik-Schüller & Lijinsky, 1981; Reddy & Lalwani, 1983). With hypolipidaemic compounds there is a good correlation between the potency of peroxisome proliferation and hepatocarcinogenicity. Because of this well established association, it is reasonable to assume that evaluation of induction of peroxisome proliferation will serve as a useful and practical test to screen non-genotoxic chemicals and also to predict their carcinogenic effect in different species of animals. The results of the experiment further support this notion because of the development of hepatocellular tumours in this highly tumour-resistant strain of mice correlates well with the peroxisome proliferation observed in short-term experiment.

This research was supported by NIH grants CA-36130 and GM-23750.

**References**

AGARWAL, D.K., LAWRENCE, W.H., NUNEZ, L.J. & AUTIAN, J. (1985). Mutagenicity evaluation of phthalic acid esters and metabolites. *J. Toxicol. Environ. Health*, **16**, 61.

BUTTERWORTH, B.E., BERMUDEZ, E., SMITH-OLIVER, T. & 7 others (1984). Lack of genotoxic activity of di(2-ethylhexyl)phthalte (DEHP) in rat and human hepatocytes. *Carcinogenesis*, **5**, 1329.

COHEN, A.J. & GRASSO, P. (1981). Review of the hepatic response to hypolipidemic drugs in rodents and assessment of its toxicological significance to man. *Food Cosmet. Toxicol.*, **19**, 585.

EACHO, P.I., FOXWORTHY, P.S., JOHNSON, W.D., HOOVER, D.M. & WHITE, S.L. (1986).. Hepatic peroxisomal changes induced by a tetrazole-substituted alkoxyacetophenone in rats and comparison with other species. *Toxicol. Appl. Pharmacol.*, **83**, 430.

ELCOMBE, C.L., ROSE, M.S. & PRATT, I.S. (1985). Biochemical, histological and ultrastructural changes in rat and mouse liver following the administration of trichloroethylene: Possible relevance to species differences to hepatocarcinogenicity. *Toxicol. Appl. Pharmacol.*, **79**, 365.

ESSIGMANN, E.M. & NEWBERNE, P.M. (1981). Enzymatic alterations in mouse hepatic nodules induced by a chlorinated hydrocarbon pesticide. *Cancer Res.*, **41**, 2823.

FIALA, S., FIALA, A.E. & DIZXON, B. (1972). γ-glutamyl transpeptidase in transplantable, chemical induced rat hepatomas and 'spontaneous' mouse hepatomas. *J. Natl Cancer Inst.*, **48**, 1393.

GLAUERT, H.P., REDDY, J.K., KENNAN, W.S., SATTLER, G.L., SUBBARAO, V. & PITOT, H.C. (1984). Effect of hypolipidemic peroxisome proliferators on unscheduled DNA synthesis in cultured hepatocytes and on mutagenesis in salmonella. *Cancer Lett.*, **24**, 147.

GOEL, S.K., LALWANI, N.D., FAHL, W.E. & REDDY, J.K. (1985). Lack of co-valent binding of peroxisome proliferators nafenopin and WY-14643 to DNA *in vivo* and *in vitro*. *Toxicol. Lett.*, **24**, 37.

GRASSO, P. & HARDY, J. (1975). Strain differences in natural incidence and response to carcinogenesis. In *Mouse hepatic neoplasia*, Butler, W.H. & Newberne, P.M. (eds) p. 111. Elsevier Press: New York.

GRAY, R.H. & DE LA IGLESIA, F.A. (1984). Quantitative microscopy comparison of peroxisome proliferation by the lipid-regulating agent gemfibrozil in several species. *Hepatology*, **4**, 420.

GUPTA, R.C., GOEL, S.K., EARLEY, K., SINGH, B. & REDDY, J.K. (1985). $^{32}$P-postlabeling analysis of peroxisome proliferator-DNA adduct formation in rat liver *in vivo* and hepatocytes *in vitro*. *Carcinogenesis*, **6**, 933.

HASHIMOTO, T. (1982). Individual peroxisomal $\beta$-oxidation enzymes. *Ann. N.Y. Acad. Sci.*, **386**, 5.

HESS, R., STAÜBLI, W. & REISS, W. (1965). Nature of the hepatomegalic effect produced by ethyl-chlorophenoxyisobutyrate in the rat. *Nature*, **208**, 856.

KIMBROUGH, R.D. (1979). The carcinogenic and other chronic effects of persistent halogenated organic compounds. *Ann. N.Y. Acad. Sci.*, **320**, 415.

KLUWE, W.M., HUFF, J.E., MATTHEWS, H.B., IRWIN, R. & HASEMAN, J.K. (1985). Comparative chronic toxicities and carcinogenic potentials of 2-ethylhexyl-containing compounds in rats and mice. *Carcinogenesis*, **6**, 1577.

LAKE, B.G., GRAY, T.J.B., FOSTER, J.R., STUBBERFIELD, C.R. & GANGULLI, S.D. (1984). Comparative studies on di(2-ethylhexyl)-phathalate-induced hepatic peroxisome proliferation in the rat and hamster. *Toxicol. Appl. Pharmacol.*, **72**, 46.

LALWANI, N.D., REDDY, M.K., QURESHI, S.A. & REDDY, J.K. (1981). Development of hepatocellular carcinomas and increased peroxisomal fatty acid $\beta$-oxidation in rats fed [4-chloro-6-(2,3-xylidino)-2-pyrimidinylthio]acetic acid (WY-14643) in the semi-purified diet. *Carcinogenesis*, **2**, 645.

LALWANI, N.D., FAHL, W.E. & REDDY, J.K. (1983). Detection of a nafenopin binding protein in rat liver cytosol associated with the induction of peroxisome proliferation by hypolipidemic compounds. *Biochem. Biophys. Res. Commun.*, **116**, 383.

LALWANI, N.D., REDDY, M.K., GHOSH, S., BARNARD, S.D., MOLELLO, J.A. & REDDY, J.K. (1985). Induction of fatty acid $\beta$-oxidation and peroxisome proliferation in the liver of rhesus monkeys by DL-040, a new hypolipidemic agent. *Biochem. Pharmacol.*, **34**, 3473.

LALWANI, N.D., ALVARES, K., REDDY, M.K., REDDY, M.N., PARIKH, I. & REDDY, J.K. (1987). Peroxisome proliferator-binding protein: Identification and partial characterization of nafenopin-, clofibric acid- and ciprofibrate-binding proteins from rat liver. *Proc. Natl Acad. Sci. USA*, **84**, 5242.

LAZAROW, P.B. & DEDUVE, C. (1976). A fatty acyl-CoA oxidizing system in rat liver peroxisomes; enhancement by clofibrate, a hypolipidemic drug. *Proc. Natl Acad. Sci USA*, **73**, 2043.

LAZAROW, P.B. (1981). Assay of peroxisomal $\beta$-oxidation of fatty acids. *Meth. Enzymol.*, **72**, 315.

LIPSKY, M.M., HINTON, D.E., KLAUNIG, J.E., GOLDBLATT, P.J. & TRUMP, B.F. (1981). Biology of hepatocellular neoplasia in the mouse. II. Sequential enzyme histochemical analysis of BALB/c mouse liver during safrole-induced carcinogenesis. *J. Natl Cancer Inst.*, **67**, 377.

LOWRY, D.H., ROSEBROUGH, N.J., FARR, A.L. & RANDALL, R.J. (1951). Protein measurement with the folin phenol reagent. *J. Biol. Chem.*, **193**, 265.

LUCK, H. (1965). Catalase. In *Methods of enzymatic analysis*, Bergmeyer, H.U. (ed) pp. 885–888. Academic Press: New York.

McCANN, J., CHOL, E., YAMASAKI, E. & AMES, B.N. (1975). Detection of carcinogens as mutagens in the salmonella/microsome test: Assay of 300 chemicals. *Proc. Natl Acad. Sci. USA*, **72**, 5135.

MIYAZAWA, S., FURUTA, S., OSUMI, T. & HASHIMOTO, T. (1980). Turnover of enzymes of peroxisomal $\beta$-oxidation in rat liver. *Biochem. Biophys. Acta*, **630**, 367.

MOODY, D.E. & REDDY, J.K. (1976). Morphometric analysis of the ultrastructural changes in rat liver induced by the peroxisome proliferator SaH 42-348. *J. Cell Biol.*, **71**, 768.

MOODY, D.E., RAO, M.S. & REDDY, J.K. (1977). Mitogenic effect in mouse liver induced by a hypolipidemic drug, nafenopin. *Virch. Arch. B. Cell Path.*, **23**, 291.

MOODY, D.E. & REDDY, J.K. (1978). Hepatic peroxisome (microbody) proliferation in rats fed plasticizers and related compounds. *Toxicol. Appl. Pharmacol.*, **45**, 497.

NATIONAL TOXICOLOGY PROGRAM (1983). National toxicology program draft report abstracts on nine chemical carcinogenesis bioassays. *Chem. Regul. Rep.*, **6**, 767.

OHMORI, T., RICE, J.M. & WILLIAMS, G.M. (1981). Histochemical characteristics of spontaneous and chemically induced hepatocellular neoplasms in mice and the development of neoplasms with gamma-glutamyl transpeptidase activity during phenobarbital exposure. *Histochem. J.*, **13**, 85.

PUGH, T.D. & GOLDFARB, S. (1978). Quantitative histochemical and autoradiographic studies of hepatocarcinogenesis in rats fed 2-acetylaminofluorene followed by phenobarbital. *Cancer Res.*, **38**, 4450.

RAO, M.S., LALWANI, N.D., WATANABE, T.K. & REDDY, J.K. (1984). Inhibitory effect of antioxidants ethoxyquin and 2(3)-tert-butyl-4-hydroxyanisole on hepatic tumorigenesis in rats fed ciprofibrate, a peroxisome proliferator. *Cancer Res.*, **44**, 1072.

RAO, M.S., SUBBARAO, V. & REDDY, J.K. (1986*a*). Peroxisome proliferator-induced hepatocarcinogenesis: Histochemical analysis of ciprofibrate-induced preneoplastic and neoplastic lesions for $\gamma$-glutamyltranspeptidase activity. *J. Natl Cancer Inst.*, **77**, 951.

RAO, M.S., TATEMATSU, M., SUBBARAO, V., ITO, N. & REDDY, J.K. (1986*b*). Analysis of peroxisome proliferator-induced preneoplastic and neoplastic lesions of rat liver for placental form of glutathione s-transferase and $\gamma$-glutamyltranspeptidase. *Cancer Res.*, **46**, 5287.

RAO, M.S. & REDDY, J.K. (1987). Peroxisome proliferation and hepatocarcinogenesis. *Carcinogenesis*, **8**, 631.

RAO, M.S., USUDA, N., SUBBARAO, V. & REDDY, J.K. (1987). Absence of $\gamma$-glutamyltranspeptidase activity in neoplastic lesions induced in the liver of male F-344 rats by di(2-ethylhexyl)phthalate, a peroxisome proliferator. *Carcinogenesis*, **8**, 1347.

REDDY, J.K. & KRISHNAKANTHA, T.P. (1975). Hepatic peroxisome proliferation: Induction by two novel compounds structurally unrelated to clofibrate. *Science*, **190**, 275.

REDDY, J.K., RAO, M.S. & MOODY, D.E. (1976). Hepatocellular carcinomas in acatalasemic mice treated with nafenopin, a hypolipidemic peroxisome proliferator. *Cancer Res.*, **36**, 1211.

REDDY, J.K. & RAO, M.S. (1977). Malignant tumors in rats fed nafenopin, a hepatic peroxisome proliferator. *J. Natl Cancer Inst.*, **59**, 1645.

REDDY, J.K., RAO, M.S., AZARNOFF, D.L. & SELL, S. (1979). Mitogenic and carcinogenic effects of a hypolipidemic peroxisome proliferator [4-chloro-6-(2,3-xylidino)2-pyrimidinylthio]acetic acid (WY-14,643) in rat and mouse liver. *Cancer Res.*, **39**, 152.

REDDY, J.K., AZARNOF, D.L. & HIGNITE, C.E. (1980). Hypolipidemic peroxisome proliferators form a novel class of chemical carcinogens. *Nature*, **283**, 397.

REDDY, J.K., WARREN, J.R., REDDY, M.K. & LALWANI, N.D. (1982*a*). Hepatic and renal effects of peroxisome proliferators: Biological implications. *Ann. N.Y. Acad. Sci.*, **386**, 81.

REDDY, J.K., LALWANI, N.D., REDDY, M.K. & QURESHI, S.A. (1982*b*). Excessive accumulation of autofluorescent lipofuscin in the liver during hepatocarcinogenesis by methyl clofenapate and other hypolipidemic peroxisome proliferators. *Cancer Res.*, **42**, 259.

REDDY, J.K. & LALWANI, N.D. (1983). Carcinogenesis by hepatic peroxisome proliferators: Evaluation of the risk of hypolipidemic drugs and industrial plasticizers to humans. *CRC Rev. Toxicol.*, **12**, 1..

REDDY, J.K. SCARPELLI, D.G., SUBBARAO, V. & LALWANI, N.D. (1983). Chemical carcinogens without mutagenic activity: Peroxisome proliferators as a prototype. *Toxicol. Pathol.*, **11**, 172.

REDDY, J.K., LALWANI, N.D., QURESHI, S.A., REDDY, M.K. & MOEHLE, C.M. (1984). Induction of hepatic peroxisome proliferation in non-rodent species including primates. *Am. J. Pathol.*, **114**, 171.

REDDY, J.K. & RAO, M.S. (1986). Peroxisome proliferators and cancer: Mechanisms and implications. *Trends Pharmacol. Sci.*, **7**, 438.

REDDY, J.K., REDDY, M.K., USMAN, M.I., LALWANI, N.D. & RAO, M.S. (1986*a*). Comparison of hepatic peroxisome proliferative effect and its implication for hepatocarcinogenicity of phthalate esters, di(2-ethylhexyl)phthalate and di(2-ethylhexyl)adipate with a hypolipidemic drug. *Environ. Health Persp.*, **65**, 317.

REDDY, J.K., GOEL, S.K., NEMALI, M.R. & 8 others (1986*b*). Transcriptional regulation of peroxisomal fatty acyl-CoA oxidase and enoyl-CoA hydratase/3-hydroxyacyl-CoA dehydrogenase in rat liver by peroxisome proliferator. *Proc. Natl Acad. Sci. USA.*, **83**, 1747.

REZNIK-SCHÜLLER, H. & LIJINSKY, W. (1981). Morphology of early changes in liver carcinogenesis by methapyrilene. *Arch. Toxicol.*, **49**, 79.

RUEBNER, B.H., GERSHWIN, M.E., FRENCH, S.W., MEIERHENRY, E., DUNN, P. & HSIEH, L.S. (1984). Mouse hepatic neoplasia: Differences among strains and carcinogens. In *Mouse liver neoplasia*, Popp, J.A. (ed) pp. 115–143. Hemisphere Publishing Corp.: Washington.

RUTENBURG, A.M., KIM, H., FISCHBEIN, J.W., HANKER, J.S., WASSERKRUG, H.L. & SELIGMAN, A.M. (1969). Histochemical and ultrastructural demonstration of gamma-glutamyl transpeptidase activity. *J. Histochem. Cytochem.*, **17**, 517.

SMALL, G.M., BURDETT, K. & CONNUCK, M.J. (1985). A sensitive spectrometric assay for peroxisomal acylCoA oxidase. *Biochem. J.*, **227**, 205.

STÄUBLI, W., HESS, R. & WEIBEL, E.R. (1969). Correlated morphometric and biochemical studies on the liver cell. *J. Cell Biol.*, **42**, 92.

SVOBODA, D.J. & AZARNOFF, D.L. (1979). Tumors in male rats fed ethyl chlorophenoxyisobutyrate, a hypolipidemic drug. *Cancer Res.*, **39**, 3419.

TATEMATSU, M., KAKU, T., MEDLINE, A. & 5 others (1982). Markers of liver neoplasia – real or fictional. In *Application of biological markers to carcinogen testing*, Milman, H.A. & Sell, S. (eds) pp. 25–41. Plenum Press: New York.

TOMASZEWSKI, K.E., AGARWAL, D.K. & MELNICK, R.L. (1986). *In vitro* steady-state levels of hydrogen peroxide after exposure of male F-344 rats and female B6C3F1 mice to hepatic peroxisome proliferators. *Carcinogenesis*, **7**, 1871.

USUDA, N., REDDY, M.K., HASHIMOTO, T., RAO, M.S. & REDDY, J.K. (1988). Tissue specificity and species differences in the distribution of urate oxidase in peroxisomes. *Lab. Invest.*, **58**, 100.

WARD, J.M. (1980). Morphology of hepatocellular neoplasms in B6C3F1 mice. *Cancer Lett.*, **9**, 319.

WARREN, J.R., SIMMON, V.F. & REDDY, J.K. (1980). Properties of hypolipidemic peroxisome proliferators in the lymphocyte [$^3$H]thymidine and salmonella mutagenesis assays. *Cancer Res.*, **40**, 36.

WEIBEL, E.R. (1969). Stereological principles of morphometry in electron microscopic cytology. *Int. Rev. Cytol.*, **26**, 235.

WEISBURGER, J.H. & WILLIAMS, G.M. (1981). Carcinogen testing: Current problems and new approaches. *Science*, **214**, 401.

WILLIAMS, G.M. (1979). Liver cell culture systems for the study of hepatocarcinogenesis. In *Advances in medical oncology, research and education*, **vol. 1**, Carcinogenesis, Margison, G.P. (ed) p. 273. Pergamon Press: New York.

YAMASAKI, H., WILBOURN, J.D. & HAROUN, L. (1982). Use of data from short term tests in the evaluation of the carcinogenicity of environmental chemicals to humans. In *Mutagens in our environment*, Sorsa, M. & Vainio, H. (eds) p. 169. Alan R. Liss, Inc.: New York.